# From Bite to Recovery: Safety and Efficacy of Pan-African Polyvalent Antivenom Used for Treating Snakebites in Cameroon

**DOI:** 10.3390/toxins18020059

**Published:** 2026-01-23

**Authors:** Tatiana K. Djikeussi, Vishwas Sovani, Rogacien Kana, Lorraine G. Nekame, Awelsa Benoit, Malama Toussaint, Louabalbe P. Emmanuel, Ngu Hilmann, Baba Souley, Issaka Sali, Yaouba Daoauda, Balkissou A. Dodo, Armelle Messa, Maraimou I. Issa, Sogueba I. Maruis, Arthur Djoumessi, Nathalie Elombo, Gavli Dongoa, Gilbert Keblouabe, Yaoua Z. Aladji

**Affiliations:** 1 Department of Immunology and Parasitology, Faculty of Health Sciences, University of Buea, Buea, Cameroon; 2 Health for Africa Now, Douala, Cameroon; 3 Pharmawisdom, Thane 400604, India; 4 District Hospital, Mayo Oulo, Cameroon; 5 District Hospital, Poli, Cameroon; 6 Regional Hospital, Garoua, Cameroon; 7 District Hospital, Gashiga, Cameroon; 8 2N Pharmaceutique, Douala, Cameroon; 9 Regional Delegation of Public Health, Garoua, Cameroon

**Keywords:** snakebite envenomation, PANAF-Premium™, antivenom safety, Cameroon, Phase IV study/post-marketing surveillance

## Abstract

Snakebite envenomation (SBE) is a major public health issue in sub-Saharan Africa (SSA), particularly in Cameroon. This Phase IV, multicenter, open-label study was conducted from June 2024 to December 2024 to evaluate the safety and efficacy of PANAF-Premium™, a World Health Organization (WHO)-approved polyvalent antivenom that was introduced in Cameroon in 2023, given that prospectively gathered data and studies on this antivenom’s safety in SSA are limited. In total, 130 victims admitted to four district hospitals in North Cameroon with confirmed SBE were included. Data on envenomation syndromes, clinical outcomes, adverse events, and treatment response were recorded. *Echis* species were responsible for most bites, while clinical syndromes included hemotoxic (68.5%), cytotoxic (30.8%), and neurotoxic (0.8%) presentations. On average, victims required 3.34 vials and 5.55 days for clinical recovery. Early antivenom administration significantly reduced the number of antivenom vials required to manage the symptoms (*p* = 0.003) and hospital stay (*p* = 0.049). Seventeen patients experienced mild to moderate adverse events. Two deaths and a case of kidney injury were noted, all unrelated to antivenom use. These study findings indicate the safety and effectiveness of PANAF-Premium™ antivenom, meeting WHO performance targets. The results highlight the importance of timely antivenom administration in treating SBEs.

## 1. Introduction

Snakebite envenoming (SBE) is life threatening and is a major public health problem in many tropical countries in the developing world. SBE is observed globally but is majorly prevalent in Southeast Asia and Africa. As per annual World Health Organization (WHO) estimates, 5.4 million people suffer snakebites worldwide, with 2.7 million SBEs and 83,000–138,000 deaths [1]. In sub-Saharan Africa (SSA), the number of snakebite cases has been estimated to be 435,000–500,000 per year, with 20,000–32,000 deaths [2].

Snakebites are often under-reported, as most SBE victims often resort to locally available traditional medicine of suspect efficacy, while many others do not seek treatment at healthcare centers [3]. Given the vast size of Africa, the epidemiology differs from region to region. According to DHIS-2 surveillance data (Cameroon Ministry of Health, 2018–2022, DHIS-2 unpublished data), 10 regions in Cameroon are the most SBE-affected ones, with 97 out of 180 health districts reporting at least one suspect case. The cumulative nationwide number of snakebite cases is estimated to be 40,518, with 1056 deaths, resulting in a case fatality rate (CFR) of 2.6%. High CFRs have been observed in the Far North (2.5%), North (6%), and Adamawa (2.6%) regions. Cameroon has been reported to have 6.6 snakebite victims per 1000 inhabitants annually, with a 3% CFR. In the northern region of Cameroon, deadly carpet vipers (*Echis ocellatus*) are common, while different vipers like *Bitis gabonica*, *B. nasicornis*, and *Atheris squamigera* populate the central region. The venoms of these vipers induce local tissue destruction, leading to necrosis and amputation, or bleeding tendencies leading to hemorrhagic shock. Elapid snakes endemic to the area are *Naja melanoleuca* and *Dendroaspis jamesoni*, whose venoms induce flaccid paralysis of the cranial nerves, rapidly extending downwards to the respiratory muscles. Additionally, *N. nigricollis* venom exhibits cytotoxic effects without causing neurotoxicity [4]. Furthermore, *E. romani*, which was recently reported to branch off from *E. ocellatus*, is found in northern Cameroon and southwest Chad, where it appears to be the leading cause of SBE-associated death [5,6].

Anti-snake venom (ASV) is derived from equines and is likely to cause early or late reactions when administered to humans. Although early allergic responses after administration of other ASVs have been previously documented, prospective data and research on the safety of the recently introduced PANAF-Premium™ antivenom in SSA are lacking [7]. Despite being administered intravenously to humans since 1896, antivenoms are unique in that there are limited rigorous clinical trials which evaluate their safety and efficacy. Assessing antivenom effectiveness in the context of SBE is complicated by various challenges. These include a wide inter-individual variation in the clinical manifestations of envenoming, difficulties in accurate identification of the offending snake (unless a dead snake is brought into the hospital with its victim and can be reliably identified), and inaccessibility and logistical challenges in areas where snakebites are common. In most cases, the patient or the bystanders often narrate the circumstances surrounding the bite; however, such information is frequently unreliable for guiding species-specific antivenom therapy. Collectively, all these factors contribute to the substantial difficulties associated with the clinical evaluation of antivenoms [8].

Notably, the WHO has recommended that post-marketing surveillance (Phase IV) studies are crucial for monitoring the safety and efficacy of antivenoms. PANAF-Premium™ (Snake Venom Antiserum-Pan Africa), manufactured by Premium Serums & Vaccines Private Ltd., India, has been approved by the WHO for use in SSA [9]. Based on preclinical testing conducted by WHO, the antivenom has been recommended for treating SBEs of 24 of the most medically important snake species in Africa. Moreover, an independent study confirmed that this antivenom effectively neutralizes the venoms of its target species (such as *N. nigricollis*, *Dendroaspis polylepis*, *E. ocellatus*, and *B. arietans*) and demonstrates marked cross-reactivity against several non-target *Naja* and *Echis* species. The neutralization potency exceeded marketed claims, extending protection against additional medically important snakes [10]. The product was recently introduced in Cameroon in 2023. Given the scarcity of prospective clinical data, in this post-marketing surveillance study, we aimed to gather data systematically about the types, severity, and frequency of adverse events (AEs) recorded following ASV administration to assess the product’s effectiveness in the clinical management of SBE victims in Cameroon.

## 2. Results

### 2.1. Patient Demographics, Snake Type Distribution, Envenomation Data, and Clinical Status

**Figure 1:**
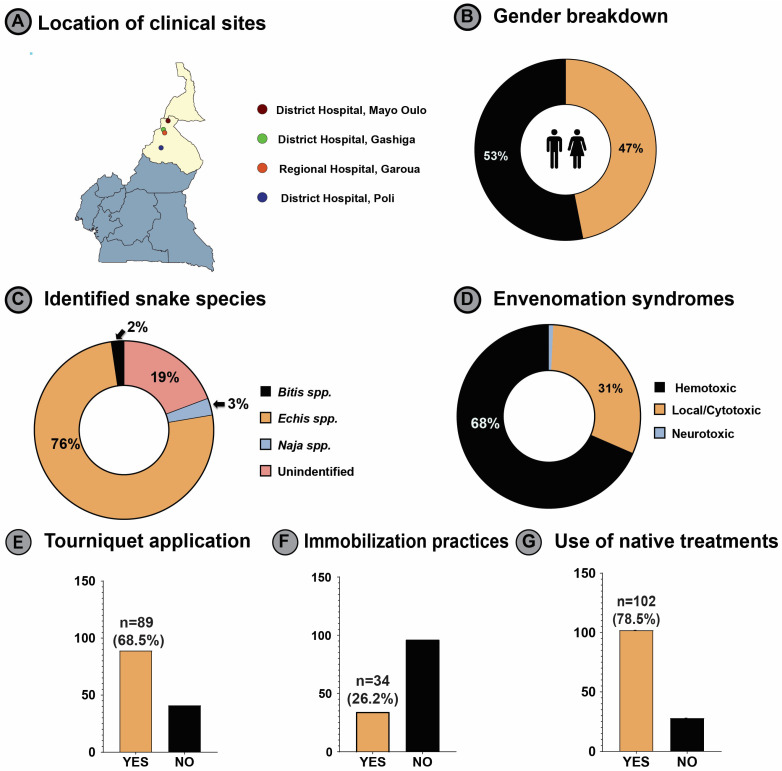
Demographic and clinical characteristics of snakebite victims. (**A**) Location of clinical sites; (**B**) gender breakdown; (**C**) identified snake species; (**D**) envenomation syndromes; (**G**) use of native treatments; (**E**) tourniquet application; (**F**) immobilization practices.

Based on the envenomation data for cases dealt with in 2022, four centers from health districts in North Cameroon were selected for our study: Regional hospital, Garoua 1; District Hospital, Poli; District Hospital, Mayo Oulo; and District Hospital, Gashiga (Figure 1A). A total of 155 victims of snakebite (or unknown bite) were reported to the four centers. Among these, 130 fulfilled the inclusion criteria and were enrolled in the study; of which, 53% (*n* = 69) were male victims (age range: 8 months to 65 years) and 47% (*n* = 61) were female victims (age range: 2 years to 70 years) (Figure 1B).

Overall, 51 SBE victims were below 18 years of age, 96 (73.8%) were agricultural laborers, and 15 (11.5%) were students. Tourniquet was used by 89 victims (68.5%), while only 34 (26.2%) victims were immobilized. Substantial number of SBE victims (102/130; 78.5%) had used some form of native treatment before visiting the healthcare facility (Figure 1E–G; Table S1).

The patient demographics, distribution of snake types, and envenomation data are shown in Table S1. Out of 130 victims, 98 (75%) were identified to be *Echis* bites, 3 were *Bitis* bites, and 4 were *Naja* bites, while the species responsible for the SBE could not be identified for 25 victims. Of the 4 *Naja* SBE victims, 3 victims developed cytotoxic syndrome, and one developed neurotoxic syndrome (Figure 1C,D; Table S1).

Since the clinical status was recorded at admission, 2 h, 6 h, 24 h, 3 days, and at discharge, envenomation was classified based on the evolution of symptoms and signs (Table S2). Clinicians compared photographs or specimens provided by the relatives of SBE victims with reference charts to identify the snake species responsible for the envenomation. However, in most cases, when neither photos nor snakes were available, the SBE victims identified the snakes on the reference charts (Table S3; Figure S1).

### 2.2. Efficacy of ASV

The distribution of different types of envenomation syndromes observed during the study is shown in Table 1. The number of vials used for various types of envenomation, duration of ASV administration, and duration of hospital stay are also listed. The number of ASV vials required for treatment was significantly higher for neurotoxic SBEs, compared with cytotoxic or hemotoxic SBEs (*p* = 0.025).

### 2.3. Safety of ASV

Out of 130 patients who received ASV, 17 patients experienced mild (12 patients) to moderate (5 patients) AEs (Table 2). A total of 31 AEs were noted, with 24 being mild AEs and 7 being moderate AEs (Table 2). Additionally, two deaths that were determined to be unrelated to ASV use are discussed in detail. The more common complaints were headache, dizziness, abdominal pain, colic, and nausea (Table 2).

During the study, two individuals died; however, none of these deaths were linked to ASV. The specific cases are detailed below:

- Patient number 14-138 (from Poli): A 20-year-old female, at 20 weeks of gestation, presented 10 h after sustaining an unknown snakebite on the lower limb. Upon arrival at the healthcare center, the patient was unconscious with a temperature of 35.4 °C, blood pressure of 102/67 mmHg, pulse of 148 beats/min, and oxygen saturation of 94%. She presented with bleeding gums, sphincter relaxation, vaginal bleeding, bleeding scars, a positive 20 min Whole Blood Clotting Test (20WBCT), delirium, and restlessness. Fetal heartbeats were detected. In the first hour, she received four vials of ASV, along with transfusion of one unit of matching blood. She experienced convulsions, necessitating sedation. The bleeding continued; therefore, she was administered two more ASV vials at 2 and 7 h timepoint post admission. Unfortunately, both the mother and the fetus died approximately 36 h after the bite. No further investigations, such as ultrasound, to identify the source of bleeding could be conducted, since this is a remote center. The investigator opined that the patient’s death could be attributed to the delay in admission.
- Patient number 11-102 (from Garoua): A 17-year-old male, bitten by an unknown snake, was admitted 3 days post-bite and presented with hemotoxic envenomation syndrome and extensive edema of the left leg. The patient was immediately administered with four vials of ASV within the first 24 h. His vital signs were recorded as blood pressure of 112/67 mm, a pulse of 83, blood glucose level of 0.94 g/L, and hemoglobin level of 4 mg/dL. He also presented with fatigue, a dry cough, sweating, and desaturation. He died 42 h after admission. Post-mortem ultrasound imaging revealed moderate ascites and pleural effusion, consistent with hemothorax as a result of massive internal bleeding.

#### 2.3.1. Safety of ASV in Pregnant SBE Victims

Given that ASV administration remains the only definitive treatment strategy to mitigate the effects of SBE, pregnant victims were not excluded from the treatment protocols. We encountered three pregnant victims during this study, all of whom were admitted to the Poli center. The cases are described below:

- Patient number 14-101: a 25-year-old female, 18 weeks pregnant, presented 7 days after an *Echis* bite and with bleeding and localized swelling, consistent with hemotoxic envenomation syndrome. She was administered one vial of an unknown brand of ASV on the day of the bite. Later, at the Poli facility, she was administered four more vials of PANAF-Premium™ polyvalent antivenom across two sessions, which began one hour after admission. She was discharged 6 days later; both the mother and child are currently in good health.
- Patient number 14-140: a 20-year-old female, 16 weeks pregnant, arrived at the Poli center immediately after an *Echis* bite. She was diagnosed with hemotoxic envenomation, as she presented with bleeding and severe edema. Prompt treatment with four vials of PANAF-Premium™ polyvalent antivenom and a 500 mL whole blood transfusion resulted in full maternal recovery and intact fetal viability. The patient was discharged in stable condition.
- The third pregnant victim was “Patient number 14-138”; both the mother and her 20-week fetus, unfortunately, succumbed to snakebite-associated complications, as detailed above.

#### 2.3.2. Occurrences of Acute Kidney Injury

A 39-year-old female (patient number 14-144) arrived at the hospital 19 h post-bite, presenting with hemotoxic syndrome following an *Echis* SBE. Upon admission, she presented with hematemesis, tumefaction, hematuria, anuria, vomiting, and signs of volume overload. Despite being fully conscious, she was hemodynamically unstable (heart rate: 93 bpm, blood pressure: 144/79 mmHg, body temperature: 36.6 °C), with pronounced pallor and leg swelling. Laboratory evaluation revealed leukocytosis, anemia (hemoglobin: 7.3 g/dL), thrombocytopenia, and severe renal impairment (urea: 4.8 g/L; creatinine: 198 mg/L). Urinalysis showed proteinuria and traces of ascorbic acid. WBCT showed delayed coagulation. The patient initially received eight doses of the PANAF-Premium™ polyvalent antivenom and three units of blood at the Poli center, after which her WBCT normalized. She was retained in the hospital for wound dressing and management of renal impairment. Eight days post-bite, due to symptoms of kidney failure such as hematuria, anuria, and vomiting, the patient was then transferred to the Garoua Regional Hospital. She was managed with four additional doses of the PANAF-Premium™ polyvalent antivenom, four units of blood, and 12 hemodialysis sessions, along with supportive therapy. Her renal function and urine output progressively improved, allowing discharge 18 days post-bite. The case highlights the importance of early reporting and ASV administration to avoid post-bite complications and to facilitate early recovery.

### 2.4. Mean Number of Vials Required to Manage Envenomation Symptoms

**Figure 2:**
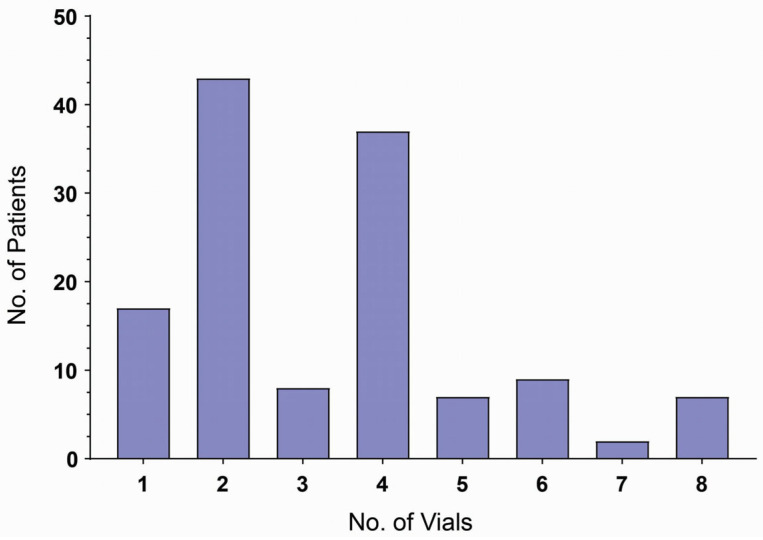
Distribution of patients by the number of antivenom vials administered.

The mean number of vials required for managing hemotoxic, local/cytotoxic, and neurotoxic envenomation symptoms was 3.45 (1.82), 2.98 (1.84), and 8.00 (-) vials, respectively (Table 1), with an overall study range of 1 to 8 vials (Figure 2). Use of six or more vials correlated with the clinical severity of edema and bleeding, both at admission and during treatment. No significant difference was observed with respect to mean vial usage across different sites, and 80% of the victims responded to four vials or less (Table S4). Notably, the Garoua Regional Hospital required the use of the highest mean number of vials (4.5).

### 2.5. Results of Bite-to-Needle Time (BNT) Analysis

The WHO performance characteristics for antivenoms are only applicable if the ASV is administered within 4–6 h of the snakebite [11]. Clinical outcomes are influenced by the delay between the time of snakebite and the time of first use of ASV is defined as the “bite-to-needle time” (BNT) [12]. The victims who received ASV within 6 h of bite, in the case of hemotoxic and local/cytotoxic envenomation, required a lesser number of vials than those who received ASV after 6 h of bite. Furthermore, the hospital stay of victims who were administered ASV early (within 6 h) was reduced by approximately 2 days, in comparison to those who received ASV after 6 h (Table 3 and Table 4). This shortened hospitalization corresponded with faster clinical recovery, as ASV administration could be stopped earlier, which resulted in shorter hospital stay, and patients received a few vials of ASV before reaching the facility. Since we could not verify the time of administration in these cases, only 123 cases have been analyzed in the present study. It revealed significant differences in vial usage across BNT more so in the cytotoxic syndromes.

## 3. Discussion

This multicenter Phase IV study provides real-world evidence of PANAF-Premium™ antivenom’s safety and efficacy for treating SBE in northern Cameroon. The predominance of hemotoxic syndromes (68.5%), primarily linked to *Echis* species (75%), is consistent with regional epidemiology and prior surveillance data. Patients treated within six hours required notably fewer vials and had shorter hospital stays compared to those treated after six hours confirming the clinical value of early intervention. The study provides real-world evidence of PANAF-Premium™ antivenom’s safety and efficacy in northern Cameroon. Our study showed that snakebite incidence in females (46.9%) was almost equal to that in males (53.1%). This result indicates a socio-demographic change, where both genders are equally affected by snakebites, contrary to the findings from earlier studies from SSA, which indicated that males were more affected by snakebites than females [13,14,15]. Tourniquets were applied in 89 cases (68.5%), while a substantial number of victims (102; 78.5%) received traditional treatment. Only in 34 cases (26.2%), the victims were immobilized (Figure 1E–G), highlighting the urgent need to raise awareness about appropriate snakebite management within the community.

The trends observed in this study shed light on the optimal treatment strategy to improve the outcomes of SBE victims, with minimal adverse effects. In most cases, appropriate clinical management requires reliable identification of a distinctive clinical syndrome, based on epidemiological, clinical, and laboratory data, which support the use of a syndromic approach, which is recommended in the majority of SBE cases [16]. The North Cameroon region is inhabited by *Echis*, *Bitis*, and *Naja* species [13,17,18]. Considering the practical difficulties in identifying snakes responsible for the SBEs, snakes were classified only at the genus level. Details of the envenomation are listed in Table 1. Alcoba et al. have mentioned that North Cameroon is populated by *Echis ocellatus*, while Central Cameroon is dominated by *Bitis* species [4]. The WHO guidelines for management of snakebites in Africa mention that bites of *Bitis* species present with extensive local swelling involving the whole limb. In West Africa, *Bitis* SBEs present with spontaneous bleeding as a result of thrombocytopenia; however, no coagulopathy is observed [19]. In the present study, all three *Bitis* SBE victims showed cytotoxicity; however, one of these victims also exhibited bleeding tendencies to the extent that envenomation had to be classified as hemotoxic envenomation. In line with WHO guidelines, envenomation by *Echis* species resulted in coagulopathy and localized effects [19]. Chippaux et al. demonstrated that 71% of *Echis* snakebite victims presented with an edema, while coagulopathy was observed in 63% of victims, and necrosis was noted in less than 5% of the victims [15]. In the present study, most victims exhibited bleeding, which was quickly controlled after ASV administration. Swelling was generally mild; however, in some victims, swelling was extensive. These victims were determined to have cytotoxic syndrome. In total, 72 of 98 (73%) *Echis* bite victims were classified to have hemotoxic envenomation and 26 to have cytotoxic envenomation. Notably, several studies have highlighted that snake venom composition is highly variable, not only between species but also among different populations of the same species from distinct geographic regions [20,21,22,23]. These phenomena could explain our discrepant findings. Among the four victims affected by *Naja* SBEs, only one victim exhibited progressive paralysis, consistent with typical neurotoxic SBE, while the other three only presented localized swelling, indicative of cytotoxic or non-neurotoxic SBE.

With regard to the number of vials required for saving SBE victims and mitigating the syndromes, Tochie et al. have reported that the management of severe snake envenomation in Cameroon requires an average of five ASV vials [13]. In a clinical trial in North Cameroon, 172 vials of antivenom were administered in 46 patients, a mean of 3.74 vials per case [9,24]. In another recent study by the same group, approximately 3–4 vials were required to mitigate the effects of SBE [9,12]. These findings are in line with those of the present study.

Further, previous studies in SSA have reported variable safety profiles for polyvalent antivenoms, with adverse event rates ranging from 10% to 30% depending on formulation and administration protocols. The performance of PANAF-Premium™ antivenom, as revealed in this study, compares favorably, particularly given the multicenter design and prospective data collection. In the present study, 17 victims experienced a total of 31 AEs, and two deaths that were determined to be unrelated to ASV administration were noted. Of the 31 AEs, 24 events were classified as mild and 7 as moderate. The more common symptoms were headache, dizziness, abdominal pain, colic, and nausea. Previous research has shown that AEs associated with SBE are not uncommon. For instance, Chippaux (1998) documented early AEs in 6.6% of SBE cases, including instances of anaphylaxis and serum sickness [25]. In a separate study, Chippaux (1999) reported that, in 46 patients with signs of SBE, 4.3% of patients experienced minor early AEs such as induration and light-headedness [24]. More recently, Chippaux et al. (2023) reported on a cohort of 447 patients who received ASV, noting that 27 had local complications at discharge, with 11 deaths, 9 cases of non-aesthetic scarring, and 2 cases of amputations [12]. In the present study, while the overall instances and types of AEs observed were similar, no cases of serum sickness or amputation were noted. Hamman et al. compared 158 cases where PANAF-Premium™ antivenom was administrated to the retrospective data collected from 6533 cases where the SBE victims received other ASVs. Among the 158 cases, 19 deaths were reported, along with 11 instances of early AEs that were classified as anaphylaxis [26]. They also reported delayed resolution of bleeding beyond 24 h. These findings are in stark contrast to our findings in similar species of snakes responsible for the SBE, despite the fact that our study was conducted in North Cameroon, not too far from the study region of the Hamman et al. study. Notably, the BTN time was 12 h in the larger subset and 24 h in the smaller one, and the doses of ASV administrated were less than those recommended in the ASV package insert, indicating deviations from the WHO-recommended treatment for SBEs using PANAF-Premium™ antivenom.

The WHO has published Target Product Profiles (TPPs) for broad-spectrum pan-African polyvalent antivenoms and has recommended a minimal and an optimal target for efficacy [11]. These optimal targets are more stringent, being exactly half of the minimum. The findings regarding the performance of the PANAF-Premium™ antivenom in comparison with the optimal performance criteria set by WHO are presented in Table 5.

Our study findings show that the performance of PANAF-Premium™ meets the requirements of optimal performance criteria for antivenoms. Based on preclinical testing, WHO had recommended the dosage of PANAF-Premium™ ASV to be used, considering the average venom yields of snake spaces [9]. As per the data of the present study, the potency and clinical efficacy PANAF-Premium™ antivenom are evident, as its administration resulted in clinical recovery at dosages lower than those recommended by the WHO.

For instance, individuals affected by hemotoxic (*Bitis* or *Echis*) envenomation, the lower end of the WHO dosage range (3–6 and 1–3 vials) was met, as the individuals required 3.45 and 2.98 vials, respectively, for clinical recovery (Table 6). Additionally, for those suffering from neurotoxic African cobra SBE, clinical recovery was achieved with eight vials. This is markedly less than the WHO recommendations of 20–40 vials (Table 6).

One of the major factors that affects the efficacy and safety of ASV is the BNT [9,12]; our findings support this. The results of the BNT analysis underscore the crucial importance of timely presentation to healthcare centers for favorable outcomes and minimizing morbidity associated with SBE. Furthermore, concerning the occurrence of AKI in one patient, it must be noted that AKI is a well-documented complication of *Echis* SBE, and prompt antivenom administration is recommended to mitigate the snakebite effects [27]. However, in this particular case, the patient sought medical attention 19 h post-bite, which is considerably delayed from the recommended timeframe for ASV administration. It is plausible that earlier administration of ASV at the healthcare center could have influenced the clinical outcome and reduced the severity of kidney damage.

The evident potent neutralizing ability of the antivenom could have been due to careful selection of immunogens, well-executed purification techniques, and/or a production process aimed at generating high-affinity, widely cross-reactive antibodies. It must be noted that such efficacy could lower the risks associated with infusions, alleviate treatment burden, and enhance cost-effectiveness in settings with limited resources. Importantly, this study is the first to directly compare PANAF-Premium™ preclinical potency data with real-life clinical outcomes. The dose requirements were lower than those recommended by the WHO based on preclinical data, highlighting the effectiveness of PANAF-Premium™ antivenom in real-world settings. A similar strategy has recently been proposed for experimental recombinant ASVs [28], and it would be highly informative to compare the real-world clinical safety and efficacy of conventional antivenoms with those of the recombinant antivenoms.

Operational gaps, including delayed presentation, reliance on native treatments, and inconsistent immobilization which must be addressed through targeted community education and first-responder’s training. Nonetheless, the findings of the present study support broader deployment of PANAF-Premium™ and underscore the need for integrated referral systems in healthcare infrastructure to reduce the bite-to-needle time. Further research should explore cost-effectiveness, long-term safety, and integration into national ASV procurement frameworks

### Limitations of the Study

However, the study has certain limitations. For instance, snake species identification relied on clinical syndromes and patient reports, which may introduce classification bias. Additionally, long-term outcomes or psychological impact were not assessed beyond 30 days. Future studies should incorporate species confirmation and extended follow-up.

## 4. Conclusions

The Pan-African PANAF-Premium™ polyvalent antivenom was demonstrated to be both safe and effective in treating SBE in North Cameroon. Our findings were similar to WHO recommendations when administered within 6–8 h of envenomation. While delayed ASV administration was still effective, it was associated with longer hospital stays and need for higher dosages.

The results of this study indicate that the syndromic approach is reliable and beneficial for managing SBE cases in resource-crunched remote areas and would enable timely and effective interventions. Lastly, expanding community outreach and awareness among the local population can help emphasize the importance of early presentation of SBE victims to healthcare facilities, which can help reduce the SBE-associated morbidity and mortality in the region.

## 5. Materials and Methods

This prospective, non-randomized, controlled, multi-centric, open-label, interventional Phase IV study was conducted from June 2024 to December 2024, after obtaining ethics committee approval from The National Ethics Committee for Human Research, Yaoundé and the Ministry of Public Health, Cameroon (Approval Number 2024/02/1638/CE/CNERSH/SP dated 14 February 2024 and ClinicalTrials.gov Identifier: NCTO6615960).

### 5.1. Inclusion Criteria

Any snakebite victim/patient, regardless of sex or age, with a history of snakebite or unknown bite, showing clinical signs of envenomation [like swelling, bleeding, unclotted 20-min Whole Blood Clotting Test (20WBCT), or signs of neuroparalysis such as blurring of vision, difficulty in swallowing, or breathing issues], necessitating the use of ASV, was included in the study.

### 5.2. Exclusion Criteria

Patients with known systemic diseases, such as pre-existing renal disease, uncontrolled chronic obstructive airway disease, congestive heart failure, or previous myocardial infarction, as well as those taking diuretics, anticoagulants, or antiplatelet drugs, were excluded. This decision was made because these pre-existing conditions and medications could potentially impact the clinical and laboratory profiles of patients with envenomation. Patients with a known history of hypersensitivity to equine-derived products, persistent substance abuse, alcohol abuse, or any physical or psychiatric condition that, as per the investigator’s judgment, could jeopardize the patient’s safety, could confound the trial results or interfere with the patient’s participation were also excluded. Pregnancy, however, was not an exclusion criterion, given that ASV is lifesaving.

### 5.3. Primary Endpoint Parameters

- Endpoint parameters for safety assessment: AEs as well as serious adverse events observed after administration of ASV were assessed for type, severity, and frequency (based on Common Terminology Criteria for Adverse Events (CTCAE) version 5, 2017) [29].
- Endpoint parameters for efficacy assessment: the number of vials of ASV used for complete clinical recovery of systemic envenomation for each syndrome, as well as the clinical outcomes (such as complete recovery, death, and amputations), were recorded.
- Other study parameters assessed included the proportion of patients presenting with envenomation, based on age, sex, syndrome, and profession, as well as the time required to control systemic toxicity and the duration of hospitalization (in days). Additionally, the types of snakes responsible for the snakebites, as identified by the patient on charts or after seeing dead snakes, were documented.

After obtaining informed consent from the patients/guardians or legally acceptable representatives, patients were enrolled for screening. Patients were clinically evaluated for the status of hemotoxic, cytotoxic, or neurotoxic syndromes at the time of admission, 2 h, 6 h, 24 h, 48 h, and at discharge. All the patients were followed up for 30 days for possible delayed reactions and/or complications to ASV.

### 5.4. Categorization of Envenomation

Envenomation was categorized based on the following signs/symptoms:

- Local/cytotoxic envenomation: presence of bite marks with or without oozing of blood, blistering, change in skin color, rapid and progressive or massive swelling involving more than half of the bitten limb within a few hours of bite (without tourniquet), and development of enlarged tender lymph nodes draining the bitten part within a couple of hours post-bite.
- Systemic envenomation:
  - For neurotoxic syndrome: signs of neuroparalysis which include blurring of vision, double vision, difficulty in swallowing, drowsiness, drooping of the head, slurring speech, unclear and indistinct voice, shallow breathing, ptosis, ataxia, respiratory paralysis, and generalized flaccid paralysis.
  - For hemotoxic syndrome: spontaneous systemic bleeding, nausea, vomiting, abdominal pain and abdominal tenderness (suggestive of gastrointestinal or retroperitoneal bleed and/or renal damage), coagulopathy detected by measuring 20WBCT with or without external bleeding, and shock.

### 5.5. Antivenom Administration

PANAF-Premium™ is a lyophilized ASV product packaged in a 20-mL glass vial, supplied with 10 mL sterile water for injection as diluent. The ASV was administered intravenously (slow infusion) over one hour. No prophylactic medications, like adrenaline, steroids, or antihistamines, were used.

**Figure 3:**
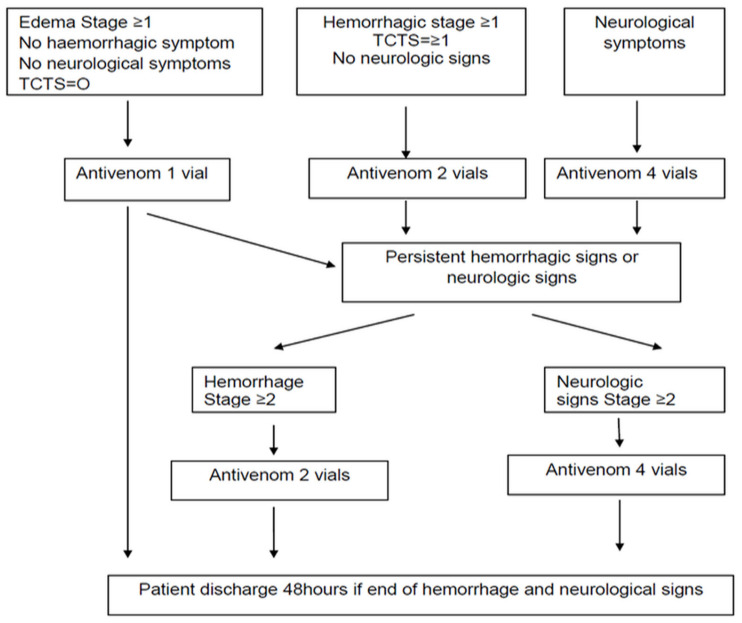
Clinical decision algorithm for antivenom administration in snakebite patients. The flowchart outlines treatment decisions based on clinical presentation. Adapted from African Society of Venimology.

In case of an AE, ASV was temporarily discontinued, and the patient was administered steroids. Once the patient had stabilized, ASV was restarted slowly, with the patient being closely monitored. Adrenaline was administered only if required. ASV was administered as per the guidance of the African Society of Venimology (Figure 3).

Demographic information, nature of snake, time of snakebite, anatomical site of bite, time interval between snakebite and ASV administration, total quantity of ASV needed, laboratory investigations, comorbid conditions, and concomitant medications were recorded on the Case Record Form.

### 5.6. BNT Analysis

To investigate BNT, the study population was divided into two groups: those who received the first ASV dose within 6 h of the bite, and those who received it later (>6 h post-snakebite). To ensure accuracy in our analysis, victims who had received ASV outside the facility before admission were excluded, as the exact timing of ASV administration and details such as the brand of ASV administered were uncertain. As a result, 123 out of 130 were found eligible for this analysis.

### 5.7. Statistical Analysis

Results were analyzed using descriptive statistics and parametric and non-parametric tests as appropriate. Statistical analysis was performed using Stata 13.1 statistical software package (Stata Corporation, College Station, TX, USA). Graphs were generated using GraphPad Prism (GraphPad Software 10.0, San Diego, CA, USA, www.graphpad.com). Demographic variables were analyzed using chi squared test, while parametric variables (such as the number of vials used, days of hospital stay, duration of ASV administration for hemotoxic and neurotoxic syndromes) were analyzed using analysis of variance (ANOVA). Results of BNT analysis were assessed using two-way ANOVA. The total analyzable population comprised 123 cases, as seven patients received ASV outside the hospital prior to admission, and we did not know the time of that injection. These had to be excluded from the analysis.

## Figures and Tables

**Table 1:** Distribution of envenomation types, number of vials of ASV used, duration of hospital stays, and duration of ASV administration (N = 130).

Envenomation Type		No. of Vials Used	Duration of ASV Administration (Hours)	Duration of Stay (Days)
	N	Mean (S.D.)	Mean (S.D.)	Mean (S.D.)
Hemotoxic	89	3.45 (1.82)	9.69 (14.15)	9.89 (14.25)
Local/Cytotoxic	40	2.98 (1.84)	9.63 (14.84)	9.19 (14.59)
Neurotoxic	1	8.00 (-)	7.02 (-)	4.00 (-)
Total	130	3.34 (1.87)	9.65 (14.25)	5.55 (8.18)
F value		3.784	0.017	0.972
*p* value		0.025	0.983	0.381

N: count; S.D.: standard deviation.

**Table 2:** Snakebite case records for patients with adverse events: symptoms, management, and outcomes.

Sr. No	Patient No.	Age	Sex	Snake Species	Symptoms	Grade	Treatment over the Stay	Outcome
1	11-102	17	M	Unknown	Cough	Moderate	Inj. Vit. K, Inj. Omeprazole, Inj. Tramadol, Inj. Tranexamic acid, blood transfusion, normal saline	No change
					Vomiting	Moderate		No change
					Sweating	Moderate		No change
					Hypotension	Moderate	Inj. Paracetamol	No change
2	12-102	21	M	*Echis*	Headache	Moderate	Nil (no information)	No change
3	12-104	33	M	Unknown	Stomach ache	Moderate	Omeprazole (I/V)	Resolved
4	12-106	30	M	Unknown	Abdominal pain	Mild	Nil (no information)	Resolved
					Nausea	Mild		Resolved
5	12-108	50	F	*Echis*	Dizziness	Mild	Inj. Dexamethasone	Resolved
6	13-101	70	F	Unknown	Stomach ache	Mild	Inj. Omeprazole	Resolved
7	13-105	33	F	*Echis*	Stomach ache	Mild	Inj. Omeprazole	Resolved
8	13-107	8	M	*Echis*	Sweating	Mild	Inj. Dexamethasone Inj. Omeprazole Inj. Metamizole	Resolved
					Restlessness	Mild		Resolved
					Headache	Mild		Resolved
9	14-101	25	F	*Echis*	Fever	Mild	Inj. Paracetamol	Resolved
10	14-109	21	F	*Naja*	Cough	Mild	Inj. Dexamethasone Inj. Paracetamol	Resolved
					Headache	Mild		Resolved
					Abdominal colic	Mild	Inj. Metoclopramide	Resolved
11	14-110	14	M	*Echis*	Headache	Mild	Inj. Paracetamol Inj. Tramadol Inj. Dexamethasone Inj. Metoclopramide	Resolved
					Itching	Moderate		Resolved
					Dizziness	Mild		Resolved
12	14-114	39	M	*Echis*	Fever	Mild	Inj. Paracetamol Inj. Tramadol	Resolved
					Headache	Mild		Resolved
13	14-115	23	M	*Echis*	Dizziness	Mild	Nil (no information)	Resolved
14	14-120	7	M	*Echis*	Headache	Mild	Inj. Paracetamol Inj. Diclofenac Inj. Metoclopramide	Resolved
					Dizziness	Mild		Resolved
15	14-121	69	F	*Echis*	Urticaria	Moderate	Inj. Paracetamol Inj. Tramadol Inj. Dexamethasone Inj. Metoclopramide	Resolved
16	14-124	24	F	*Echis*	Fever	Mild	Inj. Paracetamol	Resolved
					Headache	Mild	Inj. Tramadol	Resolved
17	14-130	16	M	*Echis*	Nausea	Mild	Inj. Tramadol,	Resolved
					Vomiting	Mild	Tab Diclofenac	Resolved

A total of 31 (23 mild and 8 moderate) AEs were observed in 17 patients.

**Table 3:** Impact of bite-to-needle time (<6 h vs. >6 h) on clinical outcomes across different envenomation types.

Number of ASV Vials Used			
**Envenomation Type**	**Time to ASV ≤ 6 h (*n*, Mean ± S.D.)**	Time to ASV > 6 h (*n*, Mean ± S.D.)	Total (*n*, Mean ± S.D.)
Hemotoxic	33, 3.00 ± 1.70	50, 3.64 ± 1.89	83, 3.38 ± 1.78
Local/Cytotoxic	16, 2.00 ± 1.15	23, 3.57 ± 1.95	39, 2.92 ± 1.83
Neurotoxic	1, 8.00	–	1, 8.00
Total	50, 2.84 ± 1.76	73, 3.62 ± 1.85	123, 3.30 ± 1.85
**Duration of Hospitalization (Days)**			
Hemotoxic	33, 3.64 ± 0.96	50, 5.92 ± 9.75	83, 5.01 ± 7.56
Local/Cytotoxic	16, 5.69 ± 7.93	23, 7.96 ± 11.05	39, 7.03 ± 9.84
Neurotoxic	1, 4.00	–	1, 4.00
Total	50, 4.30 ± 4.56	73, 6.56 ± 10.15	123, 5.64 ± 8.39

*n*: count; S.D.: standard deviation; ASV: anti-snake venom.

**Table 4:** Statistical analysis for correlation between bite-to-needle time (<6 h vs. >6 h) and clinical outcomes.

Dependent Variable	Source	Type III SS	df	Mean Square	F, *p* Value
No. of ASV Vials Used	Envenomation type	36.6	2	18.3	6.026, 0.003
	Time to ASV (≤6 h vs. >6 h)	28.6	1	28.6	9.431, 0.003
	Envenomation × Time to ASV	6.6	1	6.6	2.178, 0.143
Duration of Hospitalization	Envenomation type	107.0	2	53.5	0.759, 0.471
	Time to ASV	132.6	1	132.6	1.881, 0.173
	Envenomation × Time to ASV	0.0	1	0.0	0.000, 0.996

SS: sum of squares; df: degrees of freedom; ASV: anti-snake venom.

**Table 5:** Comparison of expected and observed clinical outcomes following timely antivenom administration (6–8 h window).

Optimal Performance Criteria: Antivenom Reduces	Current Study Findings
Case Fatality Rate < 1	No fatality *
Amputations < 1%	No amputations
Persistence of coagulopathy at 24 h post ASV administration < 3%	ASV administration was stopped within 7 h
Progression to Acute Kidney Injury (AKI) post ASV is <5%	One patient needed renal support **
Need for debridement of dead tissue and/or skin grafting (excluding decompression or deroofing of blisters) < 5%	No patient required any surgical procedure

* Two deaths were recorded during the study, which were not related to ASV. ** Patient was admitted 19 h after bite, needed 12 sessions of hemodialysis, and was discharged after 12 days.

**Table 6:** Comparison of WHO-recommended antivenom doses (based on preclinical studies) and the clinical outcomes from the present study in Cameroon.

Sr. No.	Type of Envenomation	WHO-Recommended Dosage [9]	Current Study Findings (Average Number of Vials)
1.	Hemotoxic (*Bitis* or *Echis*)	3–6 vials (For *Bitis*) 1–3 vials (For *Echis*)	3.45
2.	Local/Cytotoxic	3–6 vials (For *Bitis*) 1–3 vials (For *Echis*) 20–40 vials (African cobras)	2.98
3.	Neurotoxic	20–40 vials (African cobras)	8 (African cobras)

## Data Availability

The original contributions presented in this study are included in the article/Supplementary Materials. Further inquiries can be directed to the corresponding author.

## Supplementary Materials

The following supporting information can be downloaded at https://www.mdpi.com/article/10.3390/toxins18020059/s1, Table S1: Demographic, clinical, and snakebite characteristics across four health districts in North Cameroon (*n* = 130); Table S2: Gradation of envenomation according to the African Society of Venimology; Table S3: Summary sheet of commonly found snakes in the following habitats: Sudanese savannas, Sahelian savannas, and Mandara mountains; Table S4: Number of vials used for various patients; Figure S1: Summary sheet of images of commonly found snakes in the following habitats: Sudanese savannas, Sahelian savannas, and Mandara mountains.

## Abbreviations

The following abbreviations are used in this manuscript:

AE	Adverse events
AKI	Acute Kidney Injury
ANOVA	Analysis of variance
ASV	Anti-snake venom
BNT	Bite-to-needle time
CFR	Case fatality rate
CTCAE	Common Terminology Criteria for Adverse Events
SBE	Snakebite envenoming
SSA	Sub-Saharan Africa
TPP	Target Product Profiles
WHO	World Health Organization

## References

[1] WHO Snakebite Envenoming: A Strategy for Prevention and Control. https://apps.who.int/iris/handle/10665/324838.

[2] Gutierrez J.M., Calvete J.J., Habib A.G., Harrison R.A., Williams D.J., Warrell D.A. (2017). Snakebite envenoming. Nat. Rev. Dis. Primers.

[3] Kasturiratne A., Wickremasinghe A.R., de Silva N., Gunawardena N.K., Pathmeswaran A., Premaratna R., Savioli L., Lalloo D.G., de Silva H.J. (2008). The global burden of snakebite: A literature analysis and modelling based on regional estimates of envenoming and deaths. PLoS Med..

[4] Alcoba G., Chabloz M., Eyong J., Wanda F., Ochoa C., Comte E., Nkwescheu A., Chappuis F. (2020). Snakebite epidemiology and health-seeking behavior in Akonolinga health district, Cameroon: Cross-sectional study. PLoS Neglected Trop. Dis..

[5] Trape J.F. (2018). Partition d’Echis ocellatus Stemmler, 1970 (Squamata, Viperidae), avec la description d’une espèce nouvelle [Partitioning of Echis ocellatus Stemmler, 1970 (Squamata: Viperidae), including a description of a new species. Bull. Soc. Herpétol. Fr..

[6] Chippaux J.P., Amta P., Madec Y., Ntone R., Noel G., Clauteaux P., Boum Y., Nkwescheu A.S., Taieb F. (2025). Epidemiologic, clinical, and therapeutic aspects of formally identified Echis romani bites in northern Cameroon. PLoS Neglected Trop. Dis..

[7] Giles T., Cacala S.R., Wood D., Klopper J., Oosthuizen G.V. (2022). A retrospective study of antivenom-associated adverse reaction and anaphylaxis at Ngwelezana Hospital, South Africa. Toxicon.

[8] WHO, Health Product Policy and Standards (HPS) (2017). Guidelines for the Production, Control and Regulation of Snake Antivenom Immunoglobulins, Annex 5, TRS No 1004.

[9] WHO (2023). PANAF-Premium™ Combipack of Snake Venom Antiserum with Sterile Water for Injection (Pan Africa).

[10] Khochare S., Jaglan A., Rashmi U., Dam P., Sunagar K. (2024). Harnessing the Cross-Neutralisation Potential of Existing Antivenoms for Mitigating the Outcomes of Snakebite in Sub-Saharan Africa. Int. J. Mol. Sci..

[11] WHO (2023). Target Product Profiles for Animal Plasma-Derived Antivenoms Antivenoms for Treatment of Snakebite.

[12] Chippaux J.P., Ntone R., Benhammou D., Madec Y., Noel G., Perilhou A., Karl F., Amta P., Sanchez M., Matchim L. (2023). Real life condition evaluation of Inoserp PAN-AFRICA antivenom effectiveness in Cameroon. PLoS Neglected Trop. Dis..

[13] Tochie J.N., Temgoua M.N., Njim T., Celestin D., Tankeu R., Nkemngu N.J. (2017). The neglected burden of snakebites in Cameroon: A review of the epidemiology, management and public health challenges. BMC Res. Notes.

[14] Warrell D.A., Arnett C. (1976). The importance of bites by the saw-scaled or carpet viper (*Echis carinatus*): Epidemiological studies in Nigeria and a review of the world literature. Acta Trop..

[15] Chippaux J.P., Rage-Andrieux V., Le Mener-Delore V., Charrondiere M., Sagot P., Lang J. (2002). Epidemiology of snake envenomations in northern Cameroon. Bull. Soc. Pathol. Exot..

[16] Muller G.J., Modler H., Wium C.A., Marks C.J., Veale D.J.H. (2012). Snake bite in southern Africa: Diagnosis and management. CME Your SA J. CPD.

[17] Gonwouo N.L., LeBreton M., Chirio L., Ngassam P., Ngoa L.E., Dzikouk G. (2005). Biogeographical distribution of snakes in Cameroon: The case of venomous snakes. Bull. Soc. Pathol. Exot..

[18] Chippaux J.P., Madec Y., Amta P., Ntone R., Noel G., Clauteaux P., Boum Y., Nkwescheu A.S., Taieb F. (2024). Snakebites in Cameroon by Species Whose Effects Are Poorly Described. Trop. Med. Infect. Dis..

[19] World Health Organization, Regional Office for Africa (2010). Guidelines for the Prevention and Clinical Management of Snakebite in Africa.

[20] Ainsworth S., Menzies S.K., Casewell N.R., Harrison R.A. (2020). An analysis of preclinical efficacy testing of antivenoms for sub-Saharan Africa: Inadequate independent scrutiny and poor-quality reporting are barriers to improving snakebite treatment and management. PLoS Neglected Trop. Dis..

[21] Rashmi U., Khochare S., Attarde S., Laxme R.R.S., Suranse V., Martin G., Sunagar K. (2021). Remarkable intrapopulation venom variability in the monocellate cobra (*Naja kaouthia*) unveils neglected aspects of India’s snakebite problem. J. Proteom..

[22] Senji Laxme R.R., Khochare S., Attarde S., Suranse V., Iyer A., Casewell N.R., Whitaker R., Martin G., Sunagar K. (2021). Biogeographic venom variation in Russell’s viper (*Daboia russelii*) and the preclinical inefficacy of antivenom therapy in snakebite hotspots. PLoS Neglected Trop. Dis..

[23] Sunagar K., Undheim E.A., Scheib H., Gren E.C., Cochran C., Person C.E., Koludarov I., Kelln W., Hayes W.K., King G.F. (2014). Intraspecific venom variation in the medically significant Southern Pacific Rattlesnake (*Crotalus oreganus helleri*): Biodiscovery, clinical and evolutionary implications. J. Proteom..

[24] Chippaux J.P., Lang J., Amadi-Eddine S., Fagot P., Le Mener V. (1999). Short report: Treatment of snake envenomations by a new polyvalent antivenom composed of highly purified F(ab)2: Results of a clinical trial in northern Cameroon. Am. J. Trop. Med. Hyg..

[25] Chippaux J.P., Lang J., Eddine S.A., Fagot P., Rage V., Peyrieux J.C., Le Mener V., VAO (Venin Afrique de l’Ouest) Investigators (1998). Clinical safety of a polyvalent F(ab’)2 equine antivenom in 223 African snake envenomations: A field trial in Cameroon. Trans. R. Soc. Trop. Med. Hyg..

[26] Hamman N.A., Uppal A., Daniel E.G., Mohammed N., Nicholas N., Ballah A.S., Bappayo N., Abdulkadir B., Lawan B., Difa J.A. (2025). Epidemiology of paediatric snakebites in Northeastern Nigeria: A retrospective single-center study. BMC Pediatr..

[27] Tchaou B.A., de Tove K.S., N’Venonfon C.F.T., Mfin P.K., Aguemon A.R., Chobli M., Chippaux J.P. (2020). Acute kidney failure following severe viper envenomation: Clinical, biological and ultrasonographic aspects. J. Venom. Anim. Toxins Incl. Trop. Dis..

[28] Ahmadi S., Burlet N.J., Benard-Valle M., Guadarrama-Martinez A., Kerwin S., Cardoso I.A., Marriott A.E., Edge R.J., Crittenden E., Neri-Castro E. (2025). Nanobody-based recombinant antivenom for cobra, mamba and rinkhals bites. Nature.

[29] US Department of Health and Human Sciences (2017). Common Terminology Criteria for Adverse Events (CTCAE) v5.

[30] Djikeussi D.T. A Post Marketing study on Safety and effectiveness of Pan-African Polyvalent Antivenom used for treatment of snake bites in Cameroon. Proceedings of the Venoms & Toxins 2025.

