# Supplementary material for: From Bite to Recovery: Safety and Efficacy of Pan-African Polyvalent Antivenom Used for Treating Snakebites in Cameroon
Source: Toxins (Basel). 2026 Jan 23;18(2):59. doi: 10.3390/toxins18020059 (PMC12944739; doi:10.3390/toxins18020059)
Supplement: Supplementary file 1 [file toxins-18-00059-s001.zip › toxins-4065699-supplementary.pdf]

Table S1: Demographic, clinical, and snakebite characteristics across four health districts in Northern Cameroon (n=130).

	RH Garoua (n=27)	HD Ghasiga (n=10)	HD Mayo oulo (n=49)	HD Poli (n=44)	Total
	Mean (S.D.)	*Mean (S.D.)*	*Mean (S.D.)*	*Mean (S.D.)*	*Mean (S.D.)*
**Age**	26.11 (13.27)	31.00 (17.59)	25.29 (17.87)	28.52 (16.00)	26.99 (16.26)
**Sex**					
	**No. (%)**	**No. (%)**	**No. (%)**	**No. (%)**	**No. (%)**
Male	16 (59.3%)	7 (70.0%)	23 (46.9%)	23 (52.3%)	69 (53.1%)
Female	11 (40.7%)	3 (30.0%)	26 (53.1%)	21 (47.7%)	61 (46.9%)
**Total**	27 (100.0%)	10 (100.0%)	49 (100.0%)	44 (100.0%)	130 (100.0%)
Chi-square: 2.317; p: 0.509					
**Types of snakes**					
Bitis species	1 (3.7%)	1 (10.0%)	0 (-)	1 (2.3%)	3 (2.3%)
Echis species	17 (63.0%)	6 (60.0%)	35 (71.4%)	40 (90.9%)	98 (75.4%)
Naja species	1 (3.7%)	0 (0.0%)	2 (4.1%)	1 (2.3%)	4 (3.1%)
Unidentified	8 (29.6%)	3 (30.0%)	12 (24.5%)	2 (4.5%)	25 (19.2%)
**Total**	27 (100.0%)	10 (100.0%)	49 (100.0%)	44 (100.0%)	130 (100.0%)
Chi-square: 14.654; p: 0.101					
**Type of envenomation**					
Hemotoxic	16 (59.3%)	2 (20.0%)	35 (71.4%)	36 (81.8%)	89 (68.5%)
Local/cytotoxic	11 (40.7%)	8 (80.0%)	14 (28.6%)	7 (15.9%)	40 (30.8%)
Neurotoxic	0 (-)	0 (-)	0 (-)	1 (2.3%)	1 (0.8%)

**Total**	27 (100.0%)	10 (100.0%)	49 (100.0%)	44 (100.0%)	130 (100.0%)
Chi-square: 18.913; p: 0.004					
**Tourniquet usage**					
Yes	22 (81.5%)	6 (60.0%)	43 (87.8%)	18 (40.9%)	89 (68.5%)
No	5 (18.5%)	4 (40.0%)	6 (12.2%)	26 (59.1%)	41 (31.5%)
**Total**	27 (100.0%)	10 (100.0%)	49 (100.0%)	44 (100.0%)	130 (100.0%)
Chi-square: 8.327; p: 0.215					
**Immobilization status**					
Yes	3 (11.1%)	6 (60.0%)	15 (30.6%)	10 (22.7%)	34 (26.2%)
No	24 (88.9%)	4 (40.0%)	34 (69.4%)	34 (77.3%)	96 (73.8%)
**Total**	27 (100.0%)	10 (100.0%)	49 (100.0%)	44 (100.0%)	130 (100.0%)
**Use of native treatment**					
Yes	24 (88.9%)	6 (60.0%)	38 (77.6%)	34 (77.3%)	102 (78.5%)
No	3 (11.1%)	4 (40.0%)	11 (22.4%)	10 (22.7%)	28 (21.5%)
**Total**	27 (100.0%)	10 (100.0%)	49 (100.0%)	44 (100.0%)	130 (100.0%)
Chi-square: 3.815; p: 0.282					

Table S2: Gradation of envenomation according to the African Society of Venimology.

Stages	Edema	Hemorrhagic signs	Neurological signs
**1**	Localized edema reaching the nearest joint	Local bleeding persists for more than an hour	Anesthesia, local tingling, and pins and needles
**2**	Progressive edema affecting no more than two joints	Bleeding from the mouth, nose and scars	Profuse sweating and salivation, vomiting, miosis
**3**	Extensive edema not extending beyond the root of the limb	Ecchymosis, hematomas, purpura, phlyctenes	Eyelid drooping, abnormal vision, hearing and swallowing
**4**	Edema extending beyond the root of the limb (anasarca)	Internal hemorrhages (peritoneal, meningeal)	Respiratory distress and inability to communicate

Table S3: Summary sheet of commonly found snakes in the following habitats: Sudanese savannas, Sahelian savannas, and Mandara mountains.

Sr. No	French Name	English Translation	Scientific Name	Venomous/Non -venomous
1	Rhinotyphlops décoré	Decorated Rhinotyphlops	*Afrotyphlops decoratus*	Non-venomous
2	Rhinotyphlops ponctué ligné	Striped Spotted Rhinotyphlops	*Afrotyphlops lineolatus*	Non-venomous
3	Serpent-ver d'Adler	Adler's Worm Snake	*Myriopholis adleri*	Non-venomous
4	Boa des sables de Müller	Müller's Sand Boa	*Eryx muelleri*	Non-venomous
5	Mamba noir	Black Mamba	*Dendroaspis polylepis*	Venomous
6	Cobra égyptien	Egyptian Cobra	*Naja haje*	Venomous
7	Cobra cracheur de Kati	Kati Spitting Cobra	*Naja katiensis (syn. N. nigricollis)*	Venomous
8	Cobra cracheur à cou noir	Black-necked Spitting Cobra	*Naja nigricollis*	Venomous
9	Vipère heurtante	Puff Adder	*Bitis arietans*	Venomous
10	Vipère du Gabon	Gaboon Viper	*Bitis gabonica*	Venomous
11	Causus maculé	Spotted Night Adder	*Causus maculatus*	Venomous
12	Vipère à cornes de nez	Nose-horned Viper	*Vipera ammodytes*	Venomous
13	Vipère rhinoceros	Rhinoceros Viper	*Bitis nasicornis*	Venomous
14	Tapis ventre blanc Viper	White-bellied Carpet Viper	*Echis leucogaster*	Venomous
15	Vipère à tapis d'Afrique de l'Ouest	West African Carpet Viper	*Echis ocellatus*	Venomous
16	Vipère à écailles de scie	Saw-scaled Viper	*Echis pyramidum/ E. carinatus*	Venomous
17	Cobra des forêts	Forest Cobra	*Naja melanoleuca*	Venomous
18	Mamba vert occidental	Western Green Mamba	*Dendroaspis viridis*	Venomous
19	Mamba de Jameson	Jameson's Mamba	*Dendroaspis jamesoni*	Venomous
20	Mamba vert de l'Est	Eastern Green Mamba	*Dendroaspis angusticeps*	Venomous

<u>Table S4:</u> Number of vials used for various patients.

No. of vials	No. of patients
1	17
2	43
3	8
4	37
5	7
6	9
7	2
8	7
**Total**	**130**

Figure S1: Summary sheet of images of commonly found snakes in the following habitats: Sudanese savannas, Sahelian savannas, and Mandara mountains.

**Non-venomous snakes**	
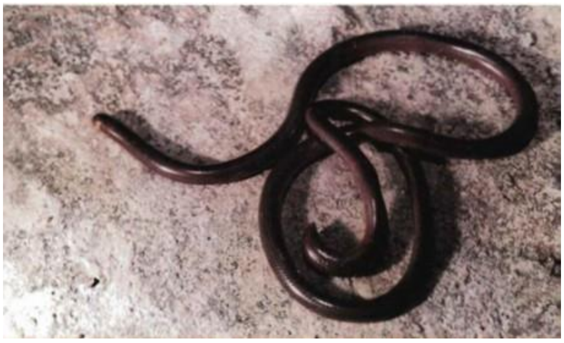	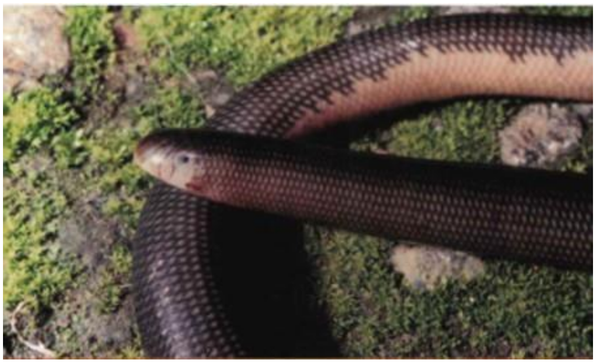
**1. Rhinotyphlops décoré**	**2. Rhinotyphlops ponctué ligné**
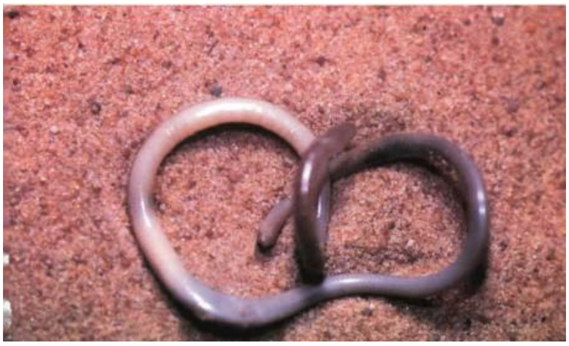	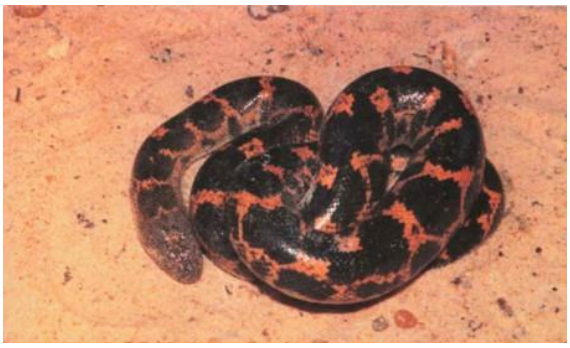
**3. Serpent-ver d'Adler**	**4. Boa des sables de Müller**
**Venomous snakes**	
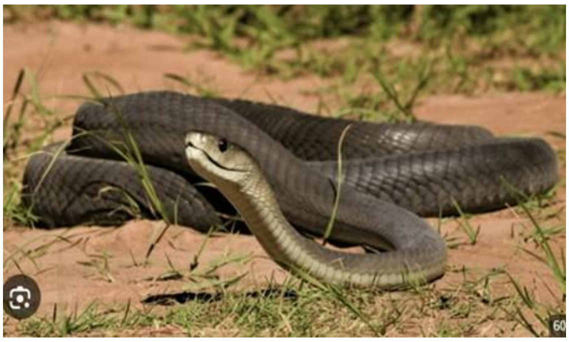	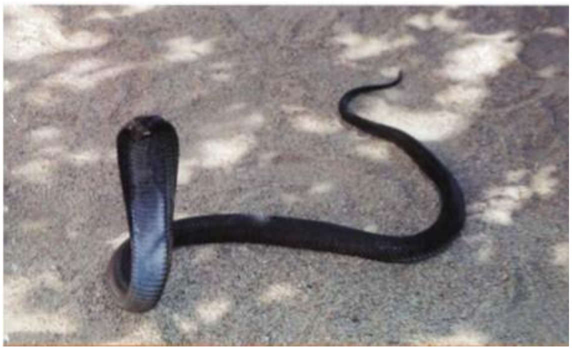
**5. Mamba noir**	**6. Cobra égyptien**

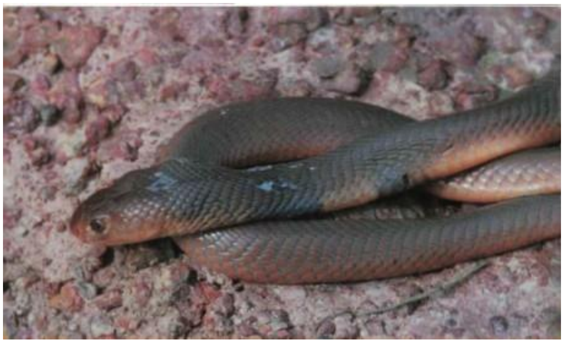	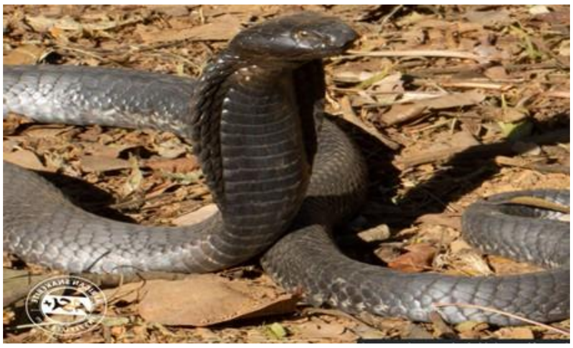
**7. Cobra cracheur de Kati**	**8. Cobra cracheur à cou noir**
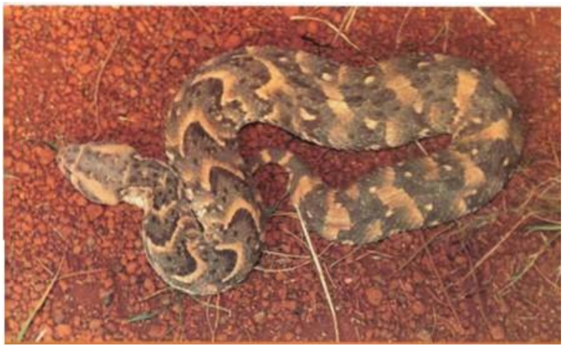	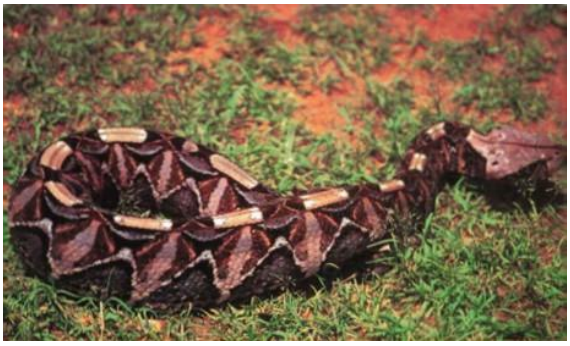
**9. Vipère heurtante**	**10. Vipère du Gabon**
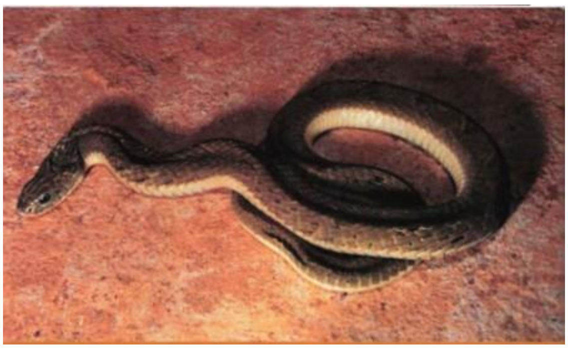	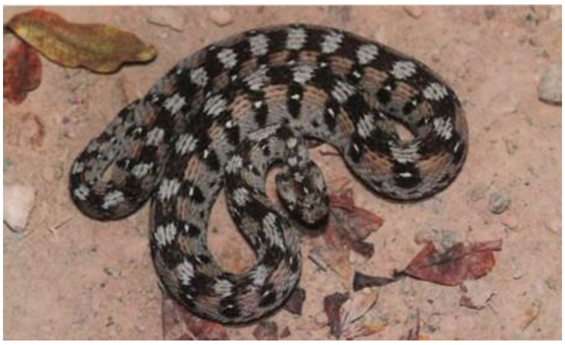
**11. Causus maculé**	**12. Vipère à cornes de nez**

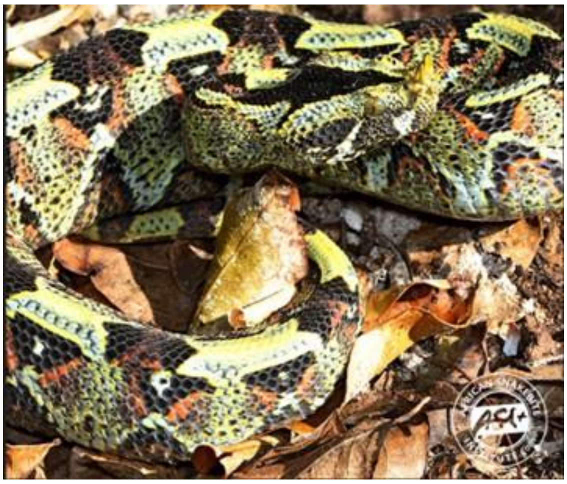	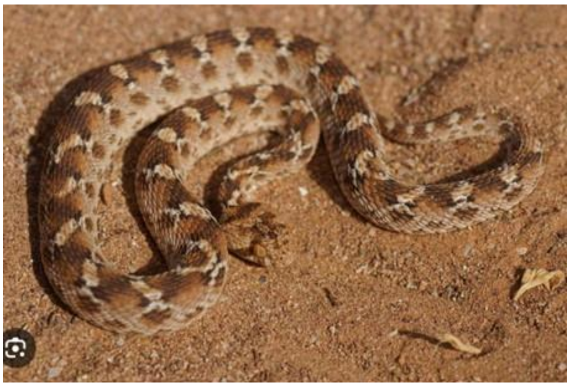
**13. Vipère rhinocéros**	**14. Tapis ventre blanc Viper**
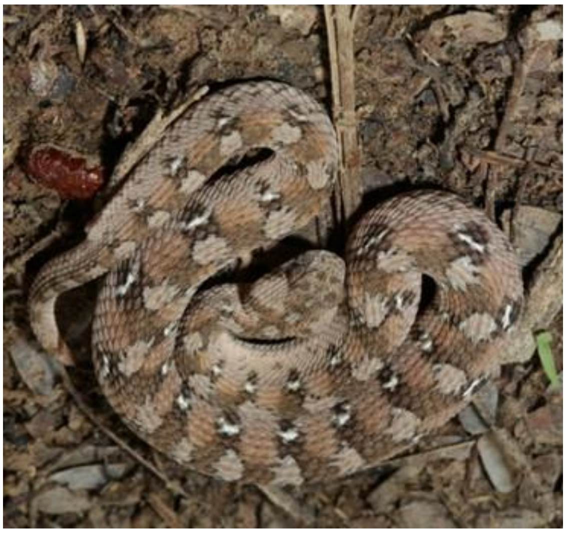	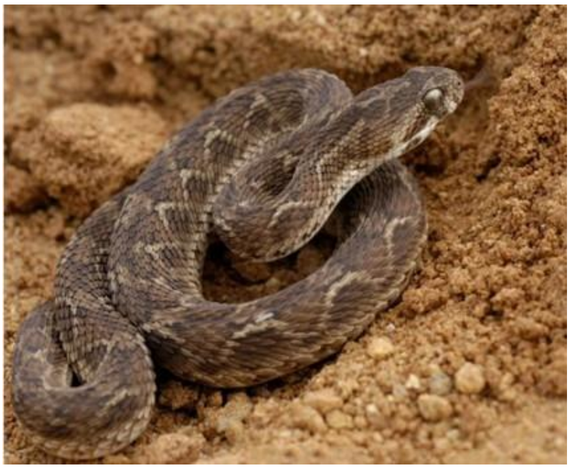
**15. Vipère à tapis d'Afrique de l'Ouest**	**16. Vipère à écailles de scie**
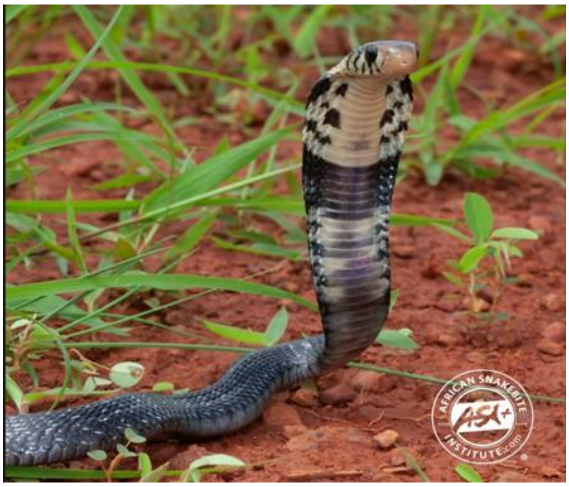	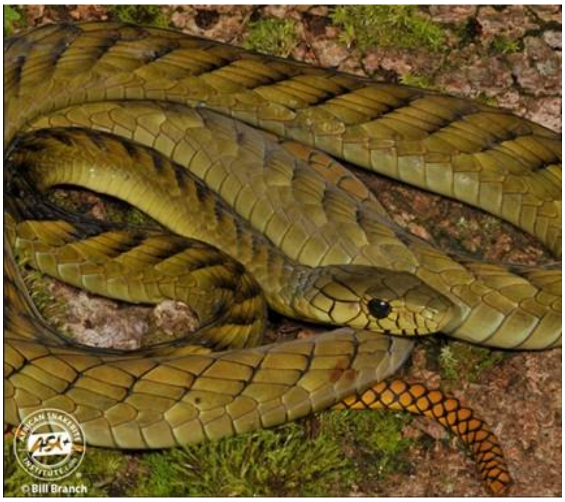
**17. Cobra des forêts**	**18. Mamba vert occidental**

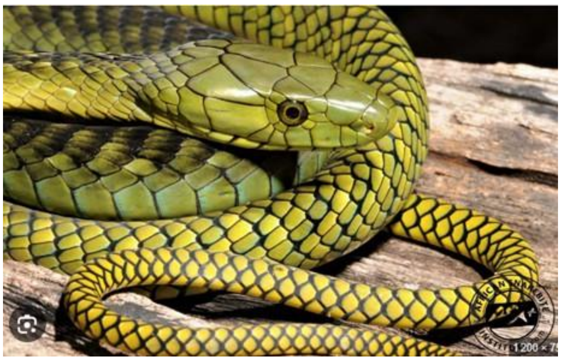	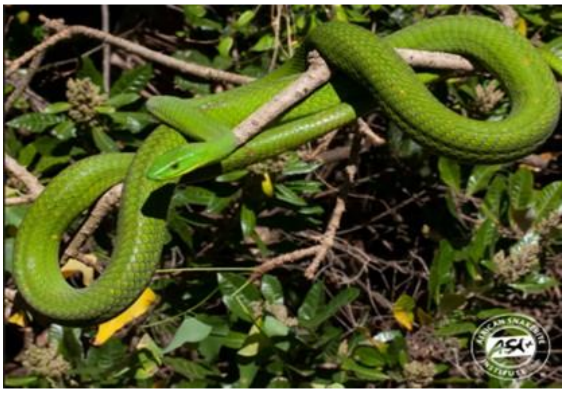
**19. Mamba de Jameson**	**20. Mamba vert de l'Est**

PLANCHE SYNTHETIQUE AVEC LES IMAGES DE SERPENTS VENIMEUX DANS LES TYPES DE FORMATIONS SUIVANTES

SAVANES SOUDANIENNES, SAVANES SAHELIENNES ET MONTS MANDARA

Note: The above chart was used to identify the snake species responsible for the bite.
